# Supplementary material for: Combination of Cannabidiol with Cisplatin or Paclitaxel Analysis Using the Chou–Talalay Method and Chemo-Sensitization Evaluation in Platinum-Resistant Ovarian Cancer Cells
Source: Biomedicines. 2025 Feb 19;13(2):520. doi: 10.3390/biomedicines13020520 (PMC11852490; doi:10.3390/biomedicines13020520)
Supplement: Supplementary file 1 [file biomedicines-13-00520-s001.zip › biomedicines-3414113-supplementary.pdf]

Supplementary Data

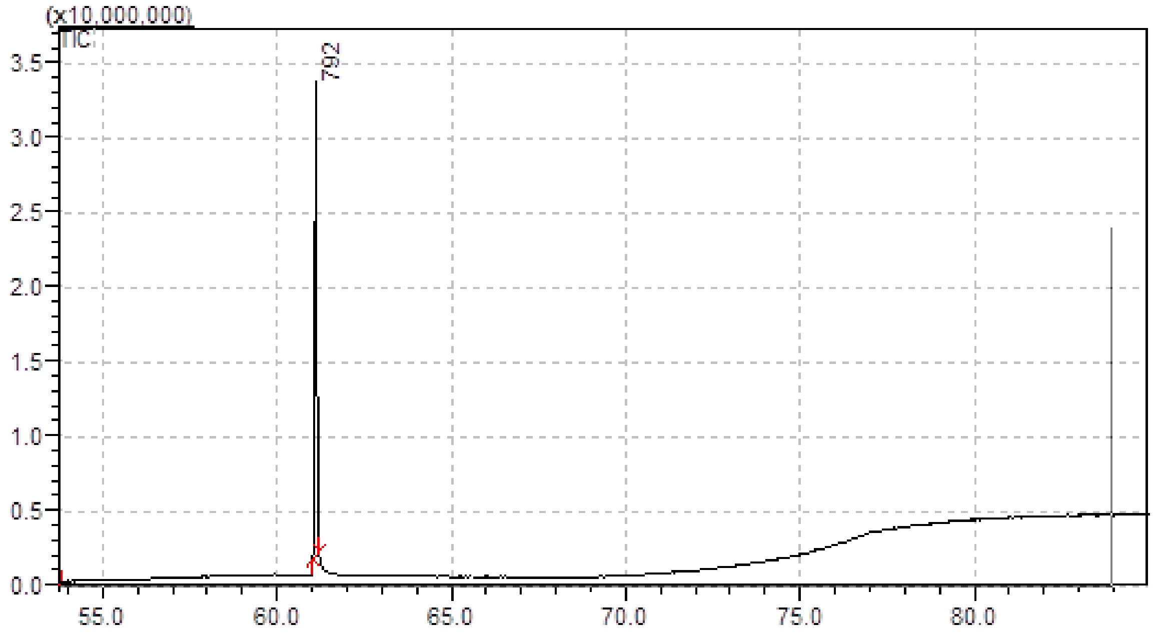

Figure S1. Total ion chromatogram (TIC) of GC-MS of the extracted CBD.

Cisplatin in Combination with CBD in ethanol:

Table S1. Inhibitory effect and Combination Indexes (CI) of the six drug combinations of CBD (in ethanol) and cisplatin performed on SK-OV-3 cell line.

Cisplatin Concentration ($\mu g\ mL^{-1}$)	CBD Concentration ($\mu g\ mL^{-1}$)	Effect (% inhibition)	CI
3.30	12.30	16	8.37
1.65	12.30	18	5.26
1.65	6.15	3	20.23
4.95	18.45	72	1.81
2.20	18.45	69	1.63
2.20	8.20	4	18.13

Table S2. Combination Indexes (CI) and DRI Data for combination of CBD (in ethanol) and cisplatin on SK-OV-3 cell line.

Fa	Cisplatin Dose (μg mL^{-1})	CBD Dose (μg mL^{-1})	CI	DRI of Cisplatin	DRI of CBD
0.05	0.18	1.99	15.74	0.08	0.26
0.10	0.37	2.96	10.06	0.15	0.31
0.15	0.57	3.77	7.72	0.20	0.36
0.20	0.81	4.52	6.36	0.26	0.39
0.25	1.06	5.26	5.44	0.32	0.43
0.30	1.36	5.99	4.77	0.37	0.46
0.35	1.69	6.76	4.24	0.47	0.49
0.40	2.09	7.56	3.81	0.53	0.52
0.45	2.54	8.42	3.44	0.62	0.55
0.50	3.09	9.35	3.12	0.71	0.58
0.55	3.75	10.39	2.84	0.83	0.61
0.60	4.58	11.57	2.58	0.96	0.65
0.65	5.63	12.93	2.34	1.12	0.69
0.70	7.03	14.58	2.12	1.32	0.73
0.75	8.98	16.63	1.89	1.59	0.79
0.80	11.87	19.33	1.68	1.95	0.85
0.85	16.64	23.20	1.46	2.51	0.94
0.90	26.06	29.57	1.22	3.52	1.07
0.95	53.80	43.74	0.92	6.04	1.32
0.97	90.12	57.78	0.77	8.89	1.53

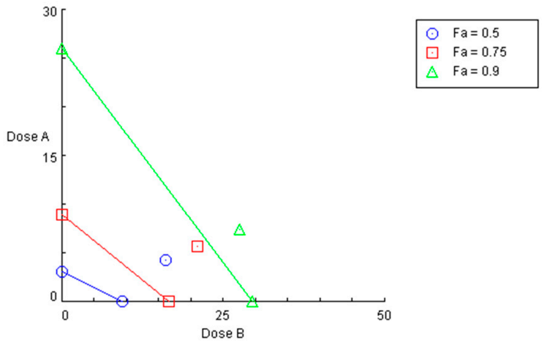

Figure S2. Isobologram for the combination of CBD (in ethanol) and cisplatin on SK-OV-3 cell line (A) (Dose A: cisplatin; Dose B: CBD). Combination data points on the diagonal line indicate an additive effect, while those on the lower left indicate synergism, and those on the upper right indicate antagonism.

Paclitaxel in Combination with CBD in ethanol:

Table S3. Error! No text of specified style in document.Inhibitory effect and Combination Indexes (CI) of the six drug combinations of CBD (in ethanol) and PTX performed on SK-OV-3 cell line.

PTX Concentration (µg mL^{-1})	CBD Concentration (µg mL^{-1})	Effect (% inhibition)	CI
9.90	12.30	33	5.69
4.95	12.30	26	5.39
4.95	6.15	27	3.98
14.85	18.45	42	5.63
6.60	18.45	23	8.86
6.60	8.20	23	6.86

Table S4. Combination Indexes (CI) and DRI Data for combination of CBD (in ethanol) and PTX on SK-OV-3 cell line.

Fa	PTX Dose (µg mL^{-1})	CBD Dose (µg mL^{-1})	CI	DRI of PTX	DRI of CBD
0.05	0.10	1.99	4.15	0.26	4.03
0.1	0.30	2.96	4.32	0.26	2.07
0.15	0.58	3.77	4.52	0.26	1.37
0.2	0.96	4.52	4.75	0.27	1.01
0.25	1.46	5.26	5.02	0.27	0.78
0.3	2.09	5.99	5.32	0.27	0.62
0.35	2.90	6.76	5.66	0.27	0.51
0.4	3.95	7.56	6.05	0.27	0.42
0.45	5.30	8.42	6.51	0.27	0.35
0.5	7.076	9.35	7.05	0.28	0.29
0.55	9.44	10.39	7.70	0.28	0.24
0.6	12.68	11.57	8.50	0.28	0.20
0.65	17.24	12.93	9.51	0.28	0.17
0.7	23.94	14.58	10.83	0.28	0.14
0.75	34.36	16.63	12.63	0.28	0.11
0.8	51.98	19.33	15.26	0.28	0.09
0.85	85.78	23.20	19.49	0.29	0.06
0.9	166.88	29.57	27.61	0.29	0.04
0.95	488.87	43.74	50.39	0.29	0.02
0.97	1050.32	57.78	78.80	0.30	0.01

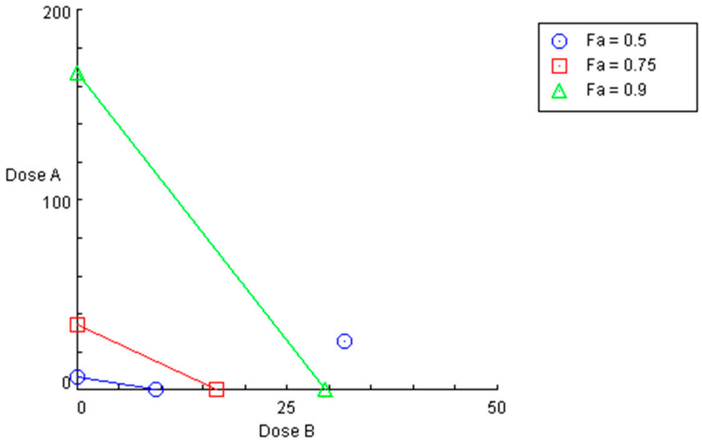

Figure S3. Isobologram for the combination of CBD (in ethanol) and PTX on SK-OV-3 cell line (Dose A: PTX; Dose B: CBD). Combination data points on the diagonal line indicate an additive effect, while those on the lower left indicate synergism, and those on the upper right indicate antagonism.
